# Protective Efficacy of VP1-Specific Neutralizing Antibody Associated with a Reduction of Viral Load and Pro-Inflammatory Cytokines in Human SCARB2-Transgenic Mice

**DOI:** 10.1371/journal.pone.0069858

**Published:** 2013-07-30

**Authors:** Hsuen-Wen Chang, Yi-Wen Lin, Hui-Min Ho, Min-Han Lin, Chia-Chyi Liu, Hsiao-Yun Shao, Pele Chong, Charles Sia, Yen-Hung Chow

**Affiliations:** 1 National Institute of Infectious Diseases and Vaccinology, National Health Research Institutes, Zhunan town, Miaoli County, Taiwan; 2 Graduate Program of Biotechnology in Medicine, Institute of Molecular Medicine, National Tsing Hua University, Hsinchu, Taiwan; 3 Graduate Institute of Immunology, China Medical University, Taichung, Taiwan; NIAID, United States of America

## Abstract

Hand-foot-mouth diseases (HFMD) caused by enterovirus 71 (EV71) and coxsackievirus 16 (CVA16) in children have now become a severe public health issue in the Asian-Pacific region. Recently we have successfully developed transgenic mice expressing human scavenger receptor class B member 2 (hSCARB2, a receptor of EV71 and CVA16) as an animal model for evaluating the pathogenesis of enterovirus infections. In this study, hSCARB2-transgenic mice were used to investigate the efficacy conferred by a previously described EV71 neutralizing antibody, N3. A single injection of N3 effectively inhibited the HFMD-like skin scurfs in mice pre-infected with clinical isolate of EV71 E59 (B4 genotype) or prevented severe limb paralysis and death in mice pre-inoculated with 5746 (C2 genotype). This protection was correlated with remarkable reduction of viral loads in the brain, spinal cord and limb muscles. Accumulated viral loads and the associated pro-inflammatory cytokines were all reduced. The protective efficacy of N3 was not observed in animals challenged with CVA16. This could be due to dissimilarity sequences of the neutralizing epitope found in CVA16. These results indicate N3 could be useful in treating severe EV71 infections and the hSCARB2-transgenic mouse could be used to evaluate the protective efficacy of potential anti-enterovirus agent candidates.

## Introduction

Enterovirus 71 (EV71) is a positive single-stranded RNA virus belonging to the Picornavirudae family. EV71 together with Coxsackievirus 16 (CVA16), CVA5, and CVA10, are known to be major causative agents that cause mild rash symptoms called hand-foot-and-mouth disease (HFMD) in infants and children [1]. Since 1997 a significant increase of EV71 epidemics has been observed throughout the Asian-Pacific region [2]. In the 1998 EV71 outbreak in Taiwan, over 100 000 young children were infected, and approximately 400 children were hospitalized with severe pulmonary and neurogenic complications that resulted in 78 deaths [3], [4].

Anti-EV71 viral drugs and vaccine are being developed, but their protective efficacy could not be efficiently evaluated due to lack of proper animal model. Several animal models have been developed to be EV71 infectious model using the mouse-adapted strain of EV71 [5], innate immunodeficient mice [6], or monkey models [7]. The intraperitoneal (i.p.) injection of clinical isolate of EV71 to adult mice caused no apparent clinical symptoms. Administration of mouse-adapted EV71 strain 4643 (Tainan/4643/98) to 1-d-old ICR mice caused hind limb paralysis (LP) and death within 2 wk of the challenge [8]. Challenge of 1-d-old BALB/C and ICR mice infected with EV71 YN3 strain was also lethal [5]. A deficiency in type I and type II IFN receptors of the AG129 mouse cause neurological manifestations after infection with the non-mouse adapted EV71 strain (5865/SIN/00009; [6]). The EV71 BrCr strain, an original prototype of the genotype A strain from California [9], was demonstrated to induce neurological manifestations of tremor, ataxia, and brain edema, but no pulmonary edema (PE) and cardiac failure in cynomolgus monkeys [7]. These models are not perfect for HFMD or for neuropathogenesis caused by EV71. Viral pathogenesis in mouse-adapted EV71-infected newborn suckling mice dose not mimic human infection and exhibit the restricted timeframe (up to one week old of mouse age) allowing for pathogenic challenge of EV71. In addition, innate immunity, particularly type I IFN, involved for the EV71-induced pathogenic phenotype was reported [10], [11].

To this end, we recently have successfully developed transgenic mice carrying the known human EV71 receptor, scavenger receptor class B member 2 (SCARB2) [11], [12]. The HFMD-like skin rashes were observed in transgenic mice pre-infected with clinical isolates E59 (genotype B4 of EV71) and N2838 (B5); severe limb paralysis and death in transgenic mice pre-inoculated with clinical isolates 5746 (C2), N3340 (C4) and coxsackievirus A16 (CVA16) [11]. EV71 viral loads in the tissues and CNS accompanied the upregulated pro-inflammatory mediators (CXCL10, CCL3, TNF-α, and IL-6), correlating to recruitment of the infiltrated T lymphocytes that resulted in severe diseases in transgenic mice [11]. It was also observed in EV71 patients with encephalitis associated with PE that have a higher mortality rate (64.3%) than patients with brainstem encephalitis (26.3%) [13], [14], PE might be caused by increased pulmonary vascular permeability resulting from brainstem lesions caused by the excessive release of IL-6, TNF-α, IL-1β, and IFN-γ [13], [15], [16]. In this study, we investigate whether the hSCARB2-transgenic mouse model could be suitable for evaluating the protective efficacy conferred by a previously described EV71-specific neutralizing antibody, N3 [17]. Administration of N3 to transgenic mice reduced the developed hair loss and scurfy skin as well as limb paralysis induced by E59 infection and protected from severe limb paralysis resulting in death by 5746 infection. This protection associates with the reduction of the viral load in the brain, spinal cords, and limb muscles. It also associated with the reduction of the secreted pro-inflammatory mediators in tissues. These results demonstrated N3 might have potential to serve as a therapeutic agent in the treatment of EV71-induced severely HFMD patients.

## Results

### EV71-specific Monoclonal Antibody N3 Cross-neutralizes E59 and 5746 Viruses

**Figure 1:**
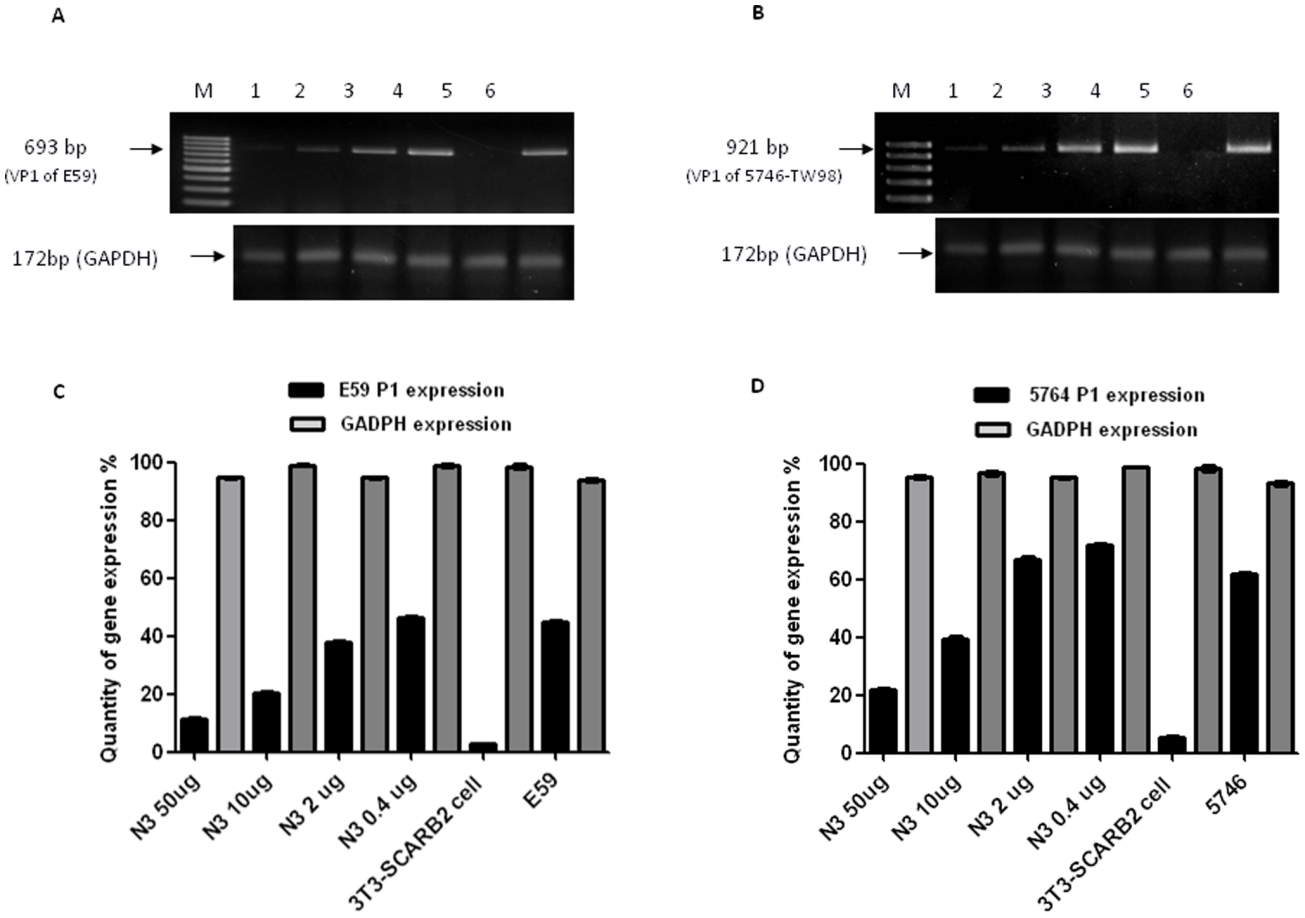
N3-mediated neutralization against B4 genotype of E59 and C2 genotype of 5746 viruses in vitro. (**A**) Both E59 and 5746 were pre-incubated (m.o.i. = 0.5) with various amounts of N3 for 1 h at 37°C before adding them to 3T3-SCARB2 cells. RNA was extracted 2 h after infection, and subjected to RT-PCR to detect the expression of viral genome P1. The amounts of N3 in different lanes were Lane 1∶50 µg, Lane 2∶10 µg, Lane 3∶2 µg, Lane 4∶0.4 µg, Lane 5: uninfected 3T3-SCARB2 cells as the negative control, and Lane 6: E59 viral cDNA as the positive control were included. The same loading scheme was applied to (**B**) where Lane 6 was replaced by 5746 virus for the control. Expression of cytosolic GADPH as the internal control of RT-PCR was detected. (**C**) and (**D**) The bar graph represents the densitometric quantification of the band intensities of viral genome P1 and GADPH from (**A**) and (**B**), respectively. The error bar of each group generated from three independent experiments was included.

To assess the capabilities of cross-neutralization of N3 against genotype B4- and C2 strains of EV71, various concentrations of N3 (beginning with 50 µg) were incubated with either E59 (B4) or 5746 (C2) before infecting NIH3T3 cells constituting expressed human SCARB2 (3T3-SCARB2) [18]. The viral genomes in infected 3T3-SCARB2 cells were detected by RT-PCR reaction using primers specifically targeting to VP1 sequence of P1 transcripts of E59 or 5746 isolates. The results show that N3 inhibited the expression of P1 of E59 and 5746 in a concentration-dependent manner. The quantities of the expressed P1, compared to the housekeeping gene glyceraldehyde-3- phosphate dehydrogenase (GAPDH) were shown in a bar graph in Fig. 1.

### N3 Conferred Protection Against E59 and 5746 Infection in hSCARB2-Transgenic Mice

**Figure 2:**
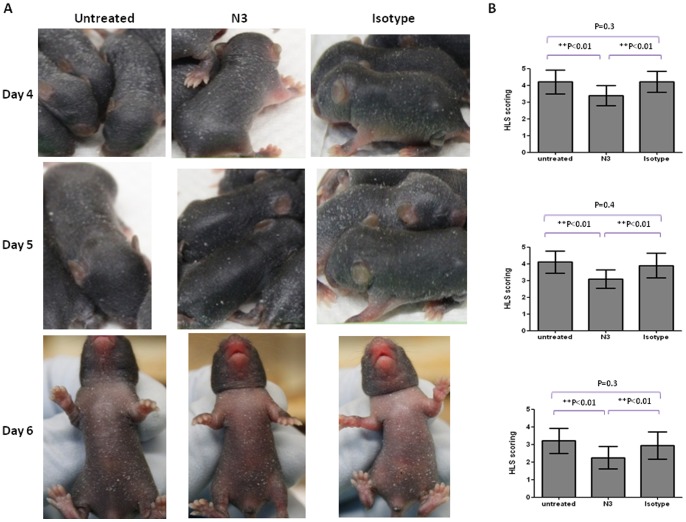
N3 conferred the reduction of hair loss and scurf on E59-preinfected hSCARB2-transgenic mice. (**A**) and (**B**) 1-day old hSCARB2-transgenic mice (n = 9–10/group) were preinfected with a HFMD symptom-causing dose of E59 at 1×10^7^ pfu subcutaneously. After 3 h of infection, 200 µg of N3 or isotype antibody was injected i.p. Mice without any antibody treatment were included. The Experiments were repeated twice, and 100× magnification of pictures (**A**) shown the representative data were taken. The graph represents the scoring of severe hair loss and skin scurf (**B**) from each group of mice. The scores were monitored on a daily basis. One-way ANOVA and the Kruskal-Wallis test were used for statistical analysis (*p< = 0.05, **p< = 0.01).

**Figure 3:**
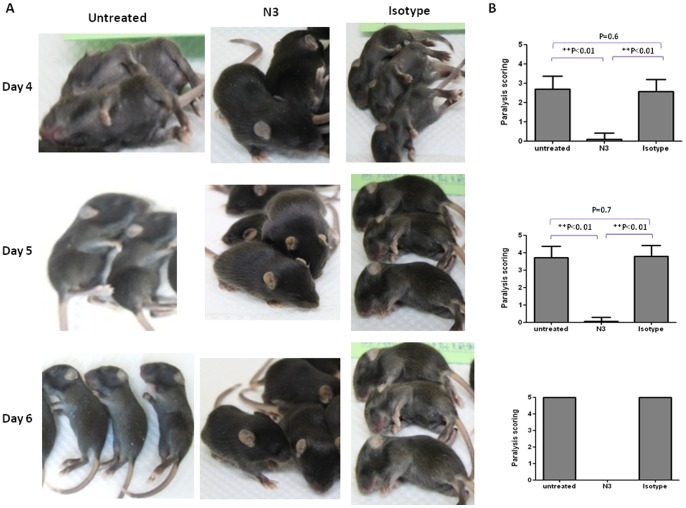
N3 conferred protection on 5746-preinfected hSCARB2-transgenic mice. (**A**) and (**B**) 7-day day old hSCARB2-transgenic mice (n = 9–10/group) were preinfected with a lethal dose of EV71 5746 virus at 1×10^5^ pfu. After 3 h of infection, 200 µg of N3 or isotype antibody was injected i.p. Mice without any antibody treatment were included. The Experiments were repeated twice, and 100× magnification of pictures (**A**) shown the representative data were taken. The graph represents the scoring of paralysis (**B**) from each group of mice. The scores were monitored on a daily basis. One-way ANOVA and the Kruskal-Wallis test were used for statistical analysis (*p< = 0.05, **p< = 0.01).

To further investigate the protective efficacy of N3 antibodies against EV71 infection in vivo, 1-day old hSCARB2-transgenic mice were preinfected with 1×10^7^ pfu of E59 subcutaneously (s.c.) followed by administration of N3 or isotype antibody (200 µg) i.p. for 3 h post infection. Mice were monitored for sign development on a daily basis. Mice treated with isotype antibody exhibited visible hair loss associated with scurfy skin peaked on Day 6 post infection, and the skin scurf/rash gradually disappeared after Day 7 post infection. Administration of N3 to E59-preinfected mice reduced the skin lesions to a relatively mild degree from Day 4 post infection onwards (Figs. 2A and 2B). Moreover, pre-challenge of 7-day old hSCARB2-transgenic mice with a lethal dose of 1×10^5^ pfu 5746 [11] caused progressive limb paralysis from Day 4 onwards and led to death if the mice remained untreated or isotype antibody treated (Figs. 3A and 3B). Mice receiving N3 treatment 3 h post infection were protected from the development of paralysis and were subsequently rescued from lethal infection.

### Timing-dependent Therapy of N3 Conferred Protection Against 5746 Infection

**Figure 4:**
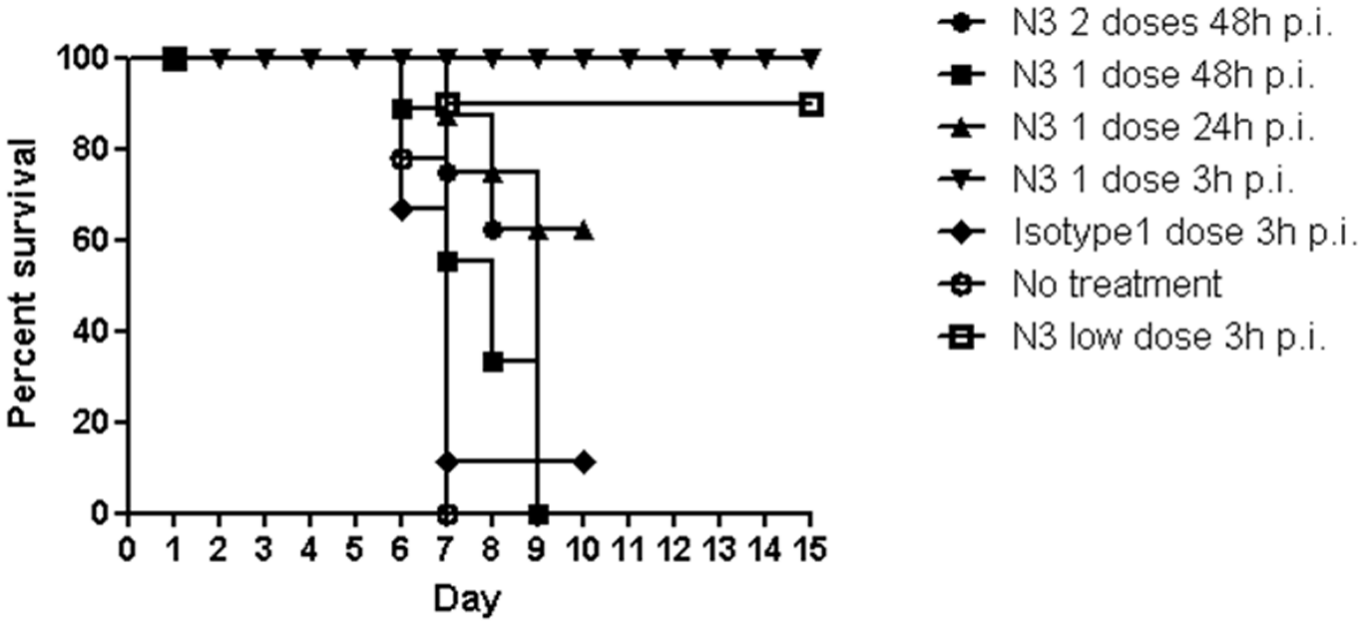
Survival of N3 treated hSCARB2-transgenic mice preinfected with 5746. Seven-day old mice preinfected with 1×10^5^ pfu of 5746 were given 200 µg N3 at time points of 3 h (▾), 24 h (▴), 48 h (▪), and 48 h twice (•) post infection. Mice injected without N3 (O), with the same amounts of isotype antibody (♦), and mice treated with a low dose of N3 (70 µg) (□) 3 h post infection were included. Mice were monitored daily and survival rates were recorded. Each group consisted of 7 to 10 mice and the results were representative of 2 independent experiments. The Logrank test was used for statistical analysis.

To investigate whether the schedule of N3 treatment is ideal for protecting from EV71 infection in vivo, 7-day old hSCARB2-transgenic mice were preinfected s.c. with a lethal dose of 1×10^5^ pfu of 5746 followed by a single dose injection of 200 µg N3 at 3, 24, or 48 h post EV71 infection, or followed by 2 doses of N3 injection (first shot at 48 h post infection followed by another 24 h later), or a low dose of N3 (70 µg) was given at 3 h post infection to examine the protection capacity. An isotype antibody was given at the same concentration (200 µg) as control in this study (Fig. 4). It was found that the mice had a better survival rate correlating to a shorter time interval of N3 administration post EV71 infection; mice were fully protected when given one dose of N3 at 3 h post infection, compared to the mice receiving N3 at 48 h post infection in which more than half of the mice died by the eighth day. Even administration of two doses of N3 at a later time (48 h post infection) could only extend the survival time period up to the eighth day post infection. Both groups of mice receiving late (48 h) treatment of N3 were died on 9 d after infection. In condition, approximately 60% of the mice receiving N3 24 h post infection were survived, and early administration of low doses of N3 3 h post infection also provided 90% protection from infection. Because early administration of 200 µg N3 might provide 100% protection against 5746 infection, this dose was chosen for following pathological studies.

### Histological Examination of 5746 Infected Mice

**Figure 5:**
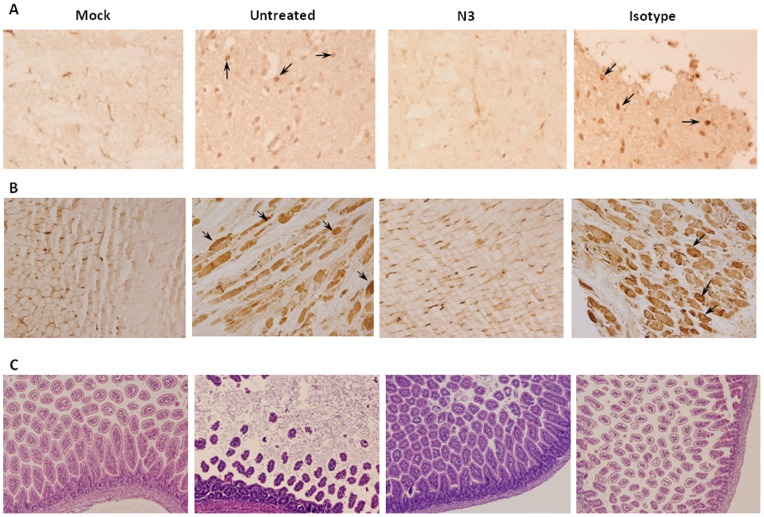
N3 reduced the pathogenesis of EV71 in the limb muscles and in the intestinal mucosa and villi. (**A**) Brainstem and (**B**) limb muscle tissues were isolated from the 1×10^5^ pfu of 5746-infected 7-day old hSCARB2-Tg mice which treated i.p. with 200 µg of N3 or isotype antibodies (3 h post infection), and from antibody-untreated mice staining with anti-EV71 antibody on Day 4 post infection. The normal tissues (MOCK) with anti-EV71 staining were also prepared. (**C**) H/E staining of intestine (>8 days post infection) from the groups of mice described in (**A**) was shown. Observations were made at a magnification of x200 for (**A**) and x100 for (**B**) and (**C**).

The pathology of EV71-infected mice treated with N3 was examined by immunohistochemical and histological staining. Mice were given 200 µg N3 or isotype antibodies at 3 h post 1×10^5^ pfu of 5746 infection. The antibody-treated animals were sacrificed and tissue sections were prepared on Day 4 after infection. No marked lesions or inflammation were observed in the brain, spinal cord, skin, heart, spleen, lungs, or liver (data not shown) of infected mice received N3 treatment. In immunochemistry staining with anti-EV71 antibody, viral particles did not accumulated in the brainstem (Fig. 5A) and muscles (Fig. 5B) of mice treated with N3, in contrast to the marked accumulation in the these tissue of mice which did not receive treatment or received only isotype antibody. The muscle fibers were damaged in the non-treated mice and mice received isotype antibody but were not observed in the tissues from the mice received N3, compared to the section of normal muscle tissue (MOCK) (Fig. 5B). Our previous study had showed that microvilli in the inner layer of the small intestine of transgenic mice would be completely destroyed after EV71 infection [11]. To investigate whether the EV71-induced pathogenesis in the intestine can be inhibited by N3 or not, histopathological stains of the intestine tissue on Day 8 post infection was performed. The result showed that the intestine from the mice received N3 was no damage, compared to massive destruction of the mucosa and microvilli in antibody-untreated or isotype antibody-treated mice (Fig. 5C).

### Detection of EV71 Expression in Different Tissues by Real-time PCR

**Figure 6:**
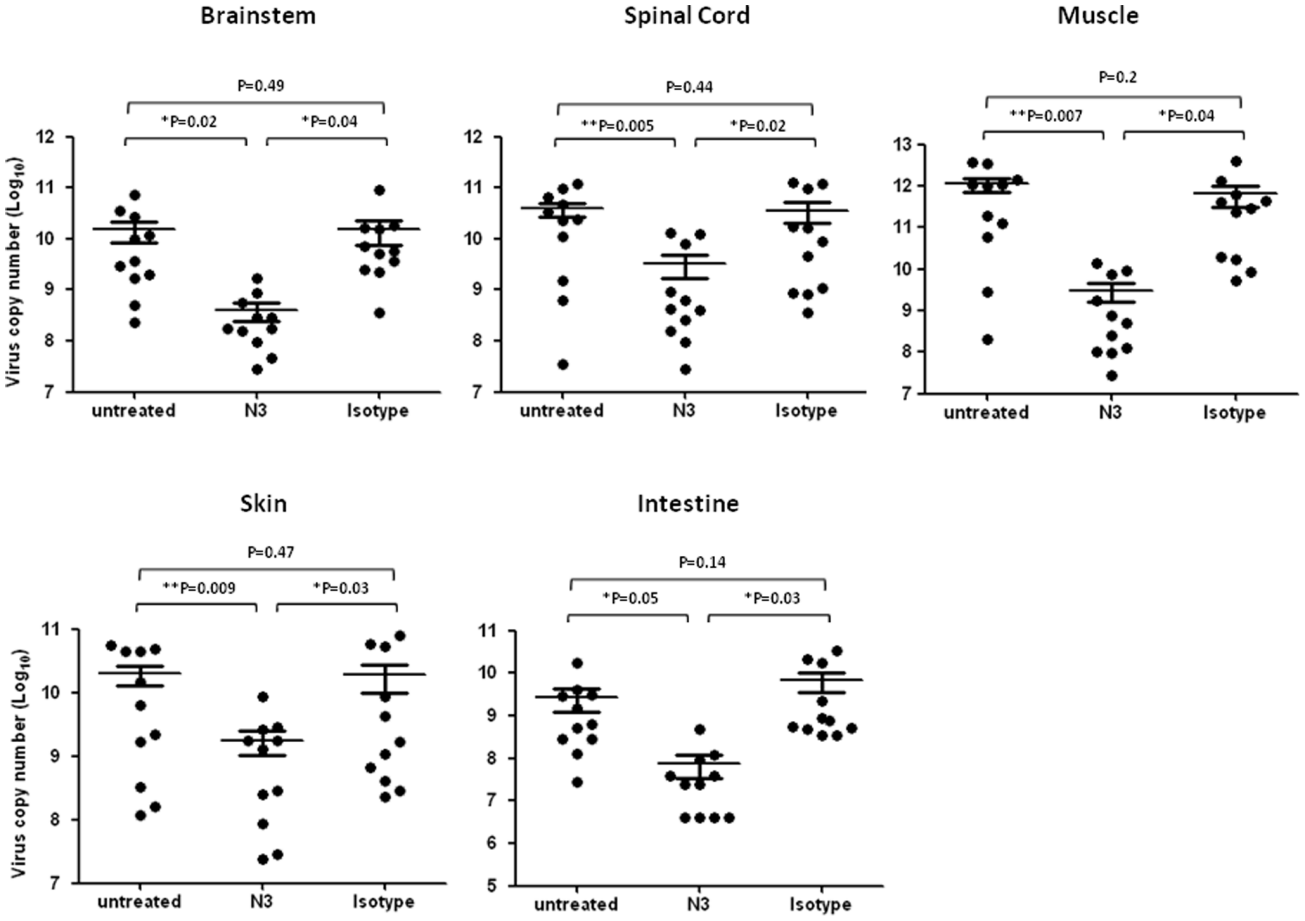
N3 inhibited viral loads of the tissues from hSCARB2-transgenic mice preinfected with EV71 5746. 7-day old hSCARB2-transgenic mice were challenged with 1×10^5^ pfu of 5746 and followed by receiving 200 µg N3 or isotype antibodies at 3 h post infection. Infected mice received no antibody were included. On day 6 post infection, mice were sacrificed and RNAs were extracted from the brainstem, spinal cord, muscle, skin, and intestine for quantitative RT-PCR using primers specific to VP1 region of P1 transcripts. Virus copy numbers were calculated using a standard curve shown by purified EV71. A schematic representation of the virus copy number and the statistical average from 11 mice per group is shown. The unpaired student *t* test with Welch correction was used for statistical analysis.

To examine the viral load of EV71 in the tissues of mice while treated with N3, we isolated RNA from the different organs from 5746-infected mice on Day 6 post infection and then quantified EV71 transcripts using real-time RT-PCR. We noticed that administration of N3 reduced viral expression significantly in the brain, spinal cord, muscle, skin, and the intestine (Fig. 6). We also confirmed that CNS- and limb muscle-tropism of EV71 viremia occurred in this model [11].

### Downregulation of Chemokines and Pro-inflammatory Cytokines after N3 Administration in 5746 Infected Mice

**Figure 7:**
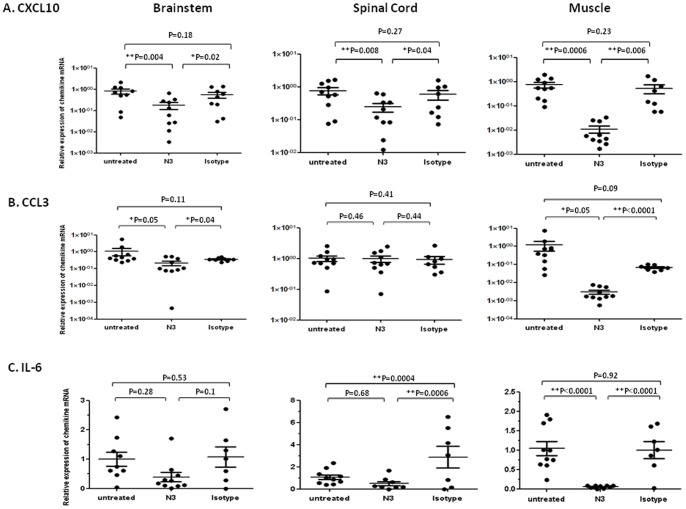
Administration of N3 reduced pro-inflammatory chemokines expression in CNS and muscle tissues of 5746-preinfected hSCARB2-transgenic mice. RNAs were extracted from the brain, spinal cord, and muscle of 5746-infected 7-day old hSCARB2-transgenic mice treated with N3 or isotype antibody, and antibody-untreated mice as described in Fig. 6 and were subjected to quantitative RT-PCR specific to (**A**) CXCL10, (**B**) CCL3, and (**C**) IL-6. The number of PCR cycles (Ct) required for fluorescent detection of target genes was calculated and presented as the relative expression after normalization with the internal control of β-actin expression from the same tissue. Each normalized 2^Ct^ value was the ratio to the value from the mean of 2^Ct^ obtained from the antibody-untreated tissues. A schematic representation of the target gene expression and the statistical average from 10 mice per group is shown. Unpaired student *t* test with Welch correction was used for statistical analysis. (*p< = 0.05, **p< = 0.01).

Upregulation of CXCL10 and CCL3 in the CNS and peripheral tissues after virulent 5746 infection in transgenic mice was reported by our previous works. This is also corresponds to the clinical findings where CXCL10 increased in patients with EV71 [19]. Higher expression of CCL3 observed in the CNS of transgenic mice infected with EV71 may contribute to the recruitment of granulocytes (neutrophils) during inflammation in the brain. Cytokine storm is thus proposed to cause severe symptoms. Administration of Intravenous immunoglobulin (IVIG) which suppresses cytokine production has been used to treat severe cases [13]–[15], [20]. We measured the expression of pro-inflammatory mediators in the tissues of 5746-infected mice while receiving N3. Quantitative RT-PCR to the specific cytokines showed that CXCL10 expression was significantly reduced in brainstem, spinal cord, and limb muscle after N3 treatment (Fig. 7A). CCL3 expression was reduced in the brain and limb muscle (Fig. 7B). Interestingly, elevated IL-6 was shown in the spinal cord but not brainstem or muscle of infected mice (untreated group vs. isotype-treated group). Treatment of N3 effectively reduced the elevated IL-6 expression in the spinal cord and even reduced the basal level of IL-6 in the muscle (Fig. 7C). These chemokines downregulated markedly in CNS and limb muscle indicated that treatment with N3 at an early stage of 5746 infection hindered viral-induced pathogenesis, and might affect the lymphocyte infiltration in the inflammatory tissues.

**Figure 8:**
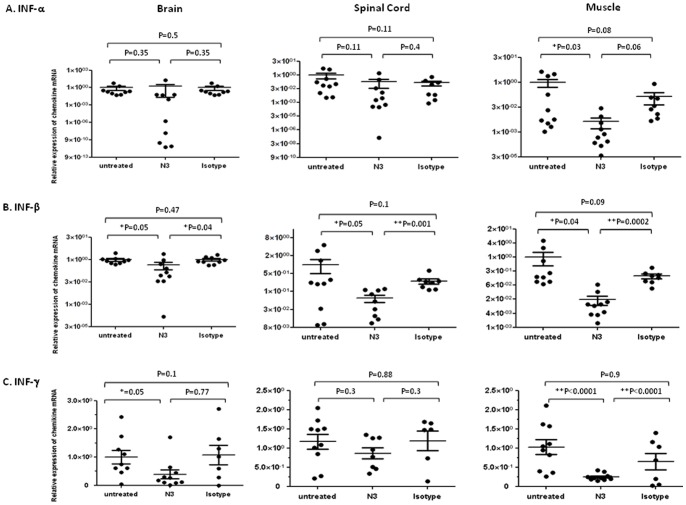
N3 downregulated type I and type II interferons in CNS and muscle tissues of 5746-infected hSCARB2-transgenic mice. RNA was extracted from the brain, spinal cord, and muscles of 5746-infected 7-day old hSCARB2-transgenic mice treated with N3 or isotype antibody, and antibody-untreated mice as described in Fig. 4 and were subjected to quantitative RT-PCR specific to (**A**) IFN-α, (**B**) IFN-β, and (**C**) IFN-γ. The relative expression of target gene normalized with the internal control of β-actin expression from the same tissue was calculated as described in Fig. 7. Unpaired student *t* test with Welch correction was used for statistical analysis. (*p< = 0.05, **p< = 0.01).

**Figure 9:**
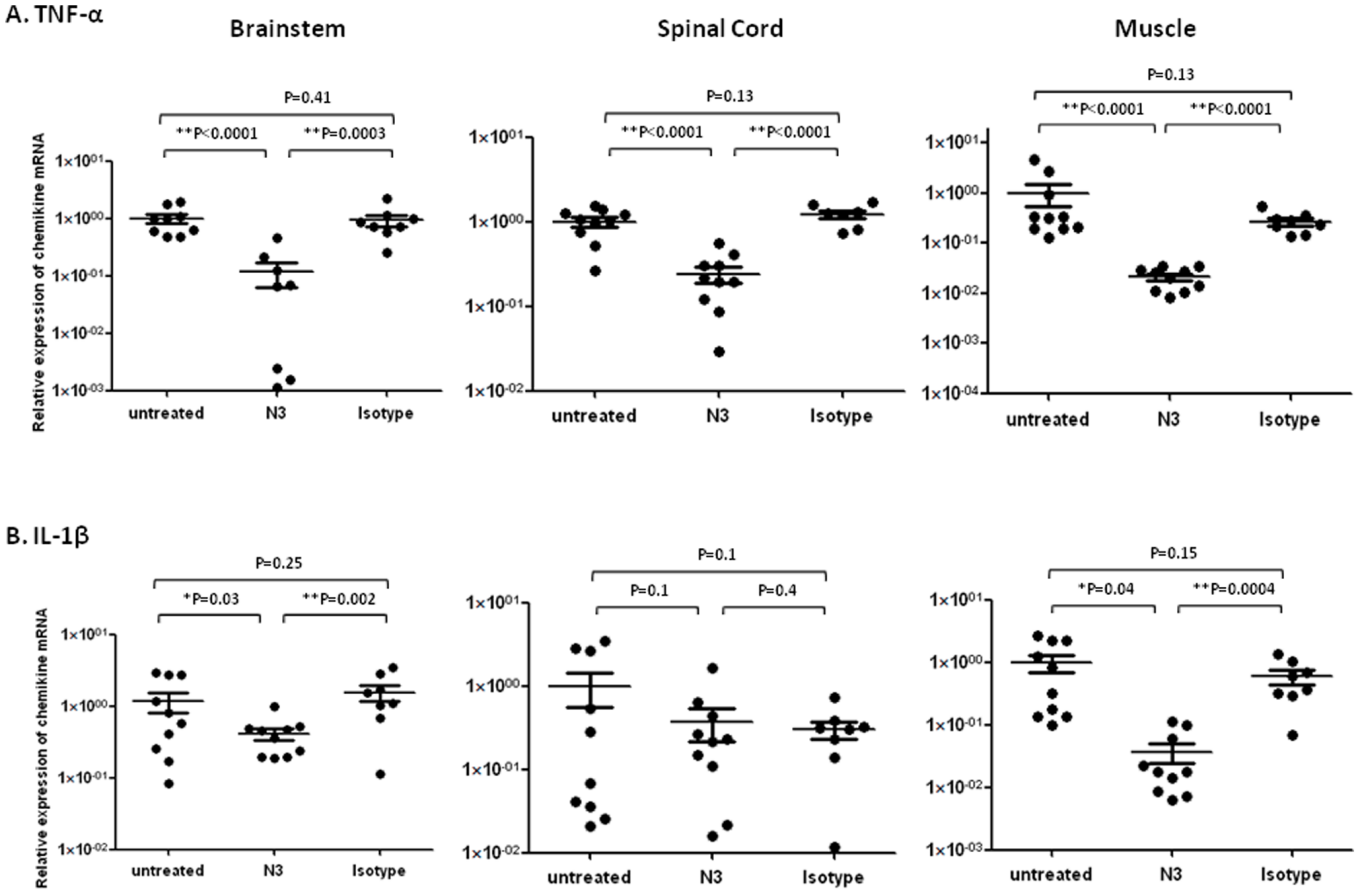
N3 downregulated TNF-α and IL-1β in CNS and muscle tissues of 5746-infected hSCARB2-transgenic mice. RNA was extracted from the brain, spinal cord, and muscles of 5746-infected 7-day old hSCARB2-transgenic mice treated with N3 or isotype antibody, and antibody-untreated mice as described in Fig. 4 and were subjected to quantitative RT-PCR specific to (**A**) TNF-α and (**B**) IL-1β. The relative expression of target gene normalized with the internal control of β-actin expression from the same tissue was calculated as described in Fig. 7. Unpaired student *t* test with Welch correction was used for statistical analysis. (*p< = 0.05, **p< = 0.01).

Similar scenarios were seen in the secretion of type I and type II interferons and pro-inflammatory cytokines in the brainstem, spinal cord, and muscle. Elevated IFN-α (muscle), IFN-β (brainstem, spinal cord, and muscle), and IFN-γ (brainstem and muscle) responding to the innate immunity against virus infection were downregulated in transgenic mice that received N3 after infection in contrast to non-treated or isotype antibody-treated mice (Fig. 8). Expression of TNF-α in three tissues and IL-1β in the brainstem and muscle of transgenic mice were also suppressed significantly (Fig. 9). These results indicate that N3 protects mice from developing pathogenesis caused by EV71 infection.

### Administration of N3 did not Protect hSCARB2-transgenic Mice from Coxsackie Virus A16 Infection

In addition to the two genotypes of EV71, we also examined whether N3 could protect hSCARB2-transgenic mice from Coxsackie virus A16 (CVA16) infection. One dose of early N3 administration after CVA16 infection significantly prolonged survival but did not rescue the mice from developing neurological syndromes which eventually led to death (Fig. S1). This result could be explained by the result of weaker cross-neutralization of N3 against CVA16 (Fig. S2). Indeed, N3 was able to recognize CVA16 virions that was evident by evaluating the binding of N3 with CVA16 using ELISA (for naïve protein interaction; Fig. S3) and Western blot (for denatured protein interaction; Fig. S4). In contrast to highly binding to two EV71 strains, N3 showed lower affinity to naïve CVA16 (Fig. S3). Oppositely, anti-CVA16 antisera, obtained from mice immunized twice with live CVA16, equally detected all EV71 virus and CVA16 particles as depicted in Fig. S4. N3 recognizes the VP1 proteins of both B4 and C2 EV71 at approximately 35 kDa [17], [21] and the VP0 of CVA16 at approximately 50 kDa [22] that is different from anti-CVA16 antisera which detected similar capsid protein patterns among both EV71 and CVA16 isolates with highlighted VP3 at approximately 26 kDa.

## Discussion

Although ICR- and BALB/C-suckling mice were reported to be susceptible to genotype C of EV71 infection to induce neurological pathology using a mouse-adapted but not clinically isolated virus in these experiments[8], [23]–[25] or using the natural strain of EV71 in type I/II interferon-deficient newborn mice [6]. The lack of a relevant animal model has hampered the development of effective prophylactic and therapeutic approaches. Previously, We had developed and demonstrated that challenge of newborn hSCARB2-transgenic mice with B genotypes of EV71 isolates, the early syndrome of HFMD, followed by progressively neurological LP were developed. In addition, hSCARB2-transgenic mice elicit more susceptibility than the reported newborn mouse models for C genotype of EV71 infection which resulted in lethal neurological diseases. Despite the muscle-tropic EV71 viremia in the reported model that differ from the CNS-tropism of EV71 infection in humans, hSCARB2-transgenic mice were characterized as bi-pathological tropism in the CNS and peripheral sites [11].

Passive transfer of protective antibodies has been shown to reduce the severity of viral infections, including Japanese encephalitis [26], varicella [27] and coxsackievirus infection [28]. Previously, we generated 4 murine monoclonal antibodies that cross-neutralized subgenogroup B isolates of EV71 in vitro. Therefore, we tested the protective efficacy of the neutralizing monoclonal antibody N3 in a reliable model. Here we show that by using N3, hSCARB2-transgenic mice infected by either genotypes B or C of EV71 viruses were protected from developing HFMD-like and paralytic symptoms. We concluded that treatment of N3 (200 µg) at an early stage (3 h post infection) after infection afforded complete protection. A lower dose (70 µg) of N3 treatment at an early stage provided 90% protection. The protection efficacy dropped when N3 was provided at 24 h post infection, even though the multiple administrations of N3 did not rescue the mice from pathogenesis. This suggested that the protection conferred by N3 is in a dose and time-dependent manner.

Several large epidemics of severe forms of EV71 infection in young children are associated with neurological diseases that may occasionally cause permanent paralysis or death [29]–[31]. In our study, we showed that N3 protected hSCARB2-transgenic mice from being infected (Fig. 4); viral load from different tissues (CNS and muscle) of N3-treated hSCARB2-transgenic mice was markedly reduced in contrast to non-treated groups (Fig. 5–6). These results indicate that the reduction of viral load in the compartment of CNS by N3 may correlate to the protection. Indeed, several studies of people and mice have also suggested the possibility of EV71-induced immunopathogenesis [19], [32]. Clinical, radiological, and laboratory features indicated that immunpathology resulting in neurological disease were reported in severe cases [33]. Elevated levels of several cytokines and chemokines have been reported in children with brainstem encephalitis and PE [13], [15]. CXCL10, CCL3, IL-6, TNF-α, IL-1β, and Type 1 and type II IFNs were found to be significantly elevated in EV71-infected hSCARB2-transgenic mice. After N3 treatment, reduction of proinflammatory mediators release was observed (Fig. 7, 8, 9). Moreover, similar results were reported in clinical cases that IFN-γ, IL-6, IL-8, IL-10, and IL-13 levels in the blood were significantly decreased in EV71 patients with PE after administration of IVIG. Beside EV71, coxsackievirus-induced myocarditis is primarily attributed to viremic injury, and immune infiltration and reclamation [34], [35]. Both innate immune response and specific host effector mechanisms were also reported to be involved in the CNS of coronavirus infection [36].

Collectively, our results show that previously generated EV71 B4 genotype monoclonal antibody, which targeted VP1 linear epitope VC43 and neutralized subgenogroups B4 and B5 strains of EV71 in vitro [17], was also able to cross-protect genotype C virus infection in vitro (Fig. 1) and in hSCARB2-transgenic mouse model. N3 conferred protection through the reduction of viral load and the secretion of proinflammatory cytokines that contributed to viremic injury and immunopathogenesis.

In contrast to the protection of N3 against the infection of EV71 in hSCARB3-transgenic mice, administration of the same amount of antibody did not protect hSCARB3-transgenic mice from lethal CVA16 infection. Investigation of the affinity of N3 against CVA16 showed lower reactivity. We have identified that N3 specifically recognized a VP1 epitope (210–225 amino acid of VP1 region or named 776–790 encoded residues of EV71 genome; [17]). In comparison of the recent study [37], alignment of the highly conserved region of VP1 amino acid (200–225) sequences from different EV71 genotypes and CVA16 showed dissimilarity between CVA16 and EV71, which may explain the scenario that N3 failed to cross-neutralize CVA16 was due to the different sequence. The epitope recognized by N3 is also different from the previous report’s epitopes; the mouse anti-sera generated from the peptides, P230–323, P646–755, P857–1012 and P1329–1440, showed strong staining with neuron plasma in both adult human cerebra and fetus medulla. N3 only closely met with the peptide P746–876 which elicits weak cross-reactivity to human tissues [38].

In conclusion, we have researched the evidence to demonstrate the cross-protection efficacy of N3 in hSCARB2-transgenic mice which show greater susceptibility to natural strains B and C in orchestrating HFMD and CNS-like syndromes and may break through the limitation of current suckling model applications that can prolong the time frame of mice age for the diseases induction especially a unique HLS syndrome [11]. This model might serve as an experimental model for aiding the evaluation of anti-EV71 therapeutic medicine. The application of humanized monoclonal antibodies to control various viral infections was reported [39], [40]. Therefore, humanized N3 is expectable to serve as therapeutic agent to treat HFMD diseases in the future. It is also of interest to further evaluate whether CVA16 could elicit cross-neutralizing antibodies against different EV71 genotypes in this model.

## Materials and Methods

### Ethics Statement

All animal experiments were conducted in accordance with the guidelines of the Laboratory Animal Center of the National Health Research Institutes (NHRI), Taiwan. The animal-use protocols were reviewed and approved by the NHRI Institutional Animal Care and Use Committee (Approved protocol no. NHRI-IACUC-101006-A). 5746-TW98 (C2) strain elicits a lethal neurological virulence of hind limb paralysis (HLP) and E59 (B4) can induce HFMD-like hair loss associated with scurf (HLS) and a non-lethal HLP in hSCARB2-transgenic mice [11]. In the protection of C2 EV71challenge, survival rate was used as one of end points to assess the protective efficacy of anti-EV71 medication. Survival rate used as an index of pathogenesis of EV71 infection in experimental animal models has been reported by many studies [5], [6], [8], [11]. In B4 challenge test, scoring of mice with HLS and HLP was performed. After the investigation or when weight loss of 20% occurred, tested animals were euthanasia by 100% CO2 inhalation for 5 minutes followed by cervical dislocation to minimize the animals suffering. To perform virus challenge, mice were explored in the anesthetic inhalator chamber containing isoflurane (For initial phase: 5%, for maintainced phase: 1.5%∼2.5%) for 1 minutes before subcutaneously or intraperitoneally immunized with EV71 or antibody, respectively.

### Viruses and Antibodies

The clinically isolated strains of EV71, genotype B4 of E59 (GenBank: GQ150746.1), was obtained from the Taiwan Centers for Diseases Control (CDC). Genotype C2 of 5746-TW98 (5746; GenBank: AF304457.1), B4 of N0781-TW-01, B5 of N2838-TW-03 (GenBank: DQ008993.1) and one strain of CVA16, 5079 (GenBank: AF177911.1) were provided by Professor Jen-Ren Wang, Department of Medical Technology, National Chen Kung University, Tainan, Taiwan. Viruses were propagated in Vero cells and purified as previously described [41], [42].

EV71-neutralizing monoclonal antibody N3 was produced in ascetic fluid and purified as previously described [17]. The specificity of N3 was checked against E59 by western blot. Commercial mouse IgG2a isotype antibody (eBioscience, CA, USA) was used as the control.

### PCR Neutralizing Assay

A PCR-based neutralization assay was adopted and performed according to Pittlot et al. [43] to evaluate the cross-neutralization of N3 against both genotypes B and C of EV71 viruses. Serial diluted N3 (10 mg/mL) were incubated with EV71 genotype B or C viruses (m.o.i. = 0.5) at 37°C for 1 h. The mixtures were then inoculated into a monolayer of the 3T3-SCARB2 cell line in a 6-well plate at 37°C for 2 h. After absorption, the cells were washed 3 times in PBS and RNA was extracted using an RNeasy kit (Qiagen, CA, USA) following instructions provided by the manufacturer. Two sets of sense and antisense synthetic oligonucleotide primers were used for the detection of EV71 genotype B and C viruses. The primer sequences used to amplify genome expending from VP1 domain of the B4 and C2 virus were 5′-AGAGAGTCACTTGCTTGGCAGACA-3′ (B4_F), and 5′-ACGACTAGTGCCGGTCGGTTTAAT-3′ (B4_R); 5′-GCTAGTGATATCCTACAGACAGGC-3′ (C2_F), 5′-GGACTGCTGTCCAAATTTCCCGAG-3′ (C2_R), respectively. The PCR was performed with a total reaction volume of 50 µl containing 2 µl of cDNA, 1× the PCR buffer with 2 mM MgCl_2_, 0.2 mM (each) of deoxynucleoside triphosphates, 0.2 µM of each primer, and 2 U of FastStart Taq polymerase (Roche, IN, USA). Amplification was conducted according to the following cycling program: denaturation at 95°C for 4 min, annealing at 52°C (B4) or 57°C (C2) for 30 s and elongation at 72°C for 3 min for 40 cycles followed by a final extension at 72°C for 7 min, all of which were performed in a 96-well Veriti Thermal Cycler (Applied Biosystem, MA, USA). The PCR products were then separated by electrophoresis in a 1.2% agarose gel containing SYBR® Safe DNA gel stain (Invitrogen, NY, USA) and visualized by ultraviolet light.

### Mouse Infection

The hSCARB2-transgemic mice were bred and maintained as previously described [11]. Both 1-day and 7-day old mice were inoculated with 1×10^7^ pfu of live E59 and 3×10^5^ pfu live 5746 virus isolates subcutaneously (s.c.). For protection studies, the purified neutralizing monoclonal antibody N3 (20 µl of 10 mg/mL) were injected intraperitoneally (i.p.) into the mice after 3, 24, and 48 h after infection. Mice were observed for symptom development for 10 days post virus infection. The severity of HFMD-like and paralysis symptoms was scored from 0 to 5 using the following criteria: For HFMD-like symptoms 5 = 80% hair loss associated with scurf (white spots) (HLS), 4 = 50% HLS, 3 = >30% HLS, 2 = >10% HLS, 1 = <10% HLS and 0 = no HLS on the back and abdomen. To score the degree of paralysis, 5 = severe paralyzed front and rear limbs with no movement, 4 = 2 rear limbs were moderately paralyzed with difficulty in movement, 3 = paralyzed rear limbs with bending legs, 2 = mildly bending rear limbs, 1 = slightly bending rear limbs, 0 = no paralyzed limbs with normal movement. The tested animals were euthanized by 100% CO2 inhalation for 5 minutes followed by cervical dislocation after completion of the experimental protocol or when weight loss of 20% occurred (according to the guidelines of approved animal-use protocols; protocol no. NHRI-IACUC-101006-A).

### Statistical Analysis

One-way ANOVA with the Kruskal-Wallis test was used to compare the results obtained from different groups with HLS or paralysis symptoms. An unpaired student *t* test with Welch correction was used to evaluate the statistical differences of the RNA expression levels between the groups. The log-rank test was used to evaluate differences in survival. A *p*-value <0.05 was considered statistically significant. The symbol * and ** were used to indicate *p*-values <0.05 and <0.01, respectively. These tests were performed using GraphPad Prism version 5.0 for Windows.

### Immunohistochemical Staining

Immunhistochemistry (IHC) was performed on paraffin-embedded tissue sections as previously described [11]. EV71 viral particles were detected using a murine anti-EV71 monoclonal antibody (Chemicon International) followed by visualization using a DAB PLUS substrate kit (Zymed Laboratories). Histopathological H/E staining was conducted in the Pathology Core Facility of the National Health Research Institute, Taiwan. Specimens were observed using a Olympus BX51 Research Microscope (model BX51TRF, Olympus, Japan). Pictures were managed using a DP controller provided by the same company.

### RNA Extraction and Quantitative Real-time PCR

Tissues from specific organs were collected in tubes containing 0.5 mL of TRIZOL reagent (Invitrogen, CA, USA) and ceramic beads (Bertin Technologies, Montigny le Bretonneux, France) followed by homogenization using Precellys 24 homogenizer (Bertin Technologies, Montigny le Bretonneux, France). RNAs were then extracted using TRIZOL reagent following the instructions provided by the manufacturer. The purity of the isolated RNAs was examined using a spectrophotometer with an A260/A280 absorbance ratio. The cDNA synthesis was performed using a Transcriptor First Strand cDNA Synthesis Kit (Roche, IN, USA) following the instructions provided by the manufacturer. One microgram of RNA was mixed with 60 µM of random hexamer primers and denatured at 65°C for 10 min before adding 1× transcriptor reverse transcriptase reaction buffer, 2 0U of RNase inhibitor, 1 mM of deoxynucleotide mix, and 1 0U of reverse transcriptase at 55°C for 30 min followed by 85°C for 5 min. The resulting cDNAs were then subjected to quantitative real-time PCR analysis with EV71 VP1 specific primers. Mouse actin genes in the samples were used as the internal control. To evaluate the expression of cytokine and chemokine genes of different tissues, specified primer pairs were used for the detection of CXCL10, CCL3, IL-6, TNF-α, INF-α, IFN-β, and IL-1β. The sequences of primer pairs specific to the target genes were the same as previously noted in [11]. To determine the expression of IFN-γ, Taqman assay was performed using specific primers 5′-TCAAAAGAGTTCCTTATGTGCCTA-3′ (Forward) and 5′- TACGAGGACGGAGAGCTGTT-3′ (Reverse) and the probe No.69 from Universal Probe Library (Cat. No. 04688686001, Roche). Maxima probe/ROX qPCR Master Mix (2X) (Cat. No. K02331, Thermo Scientific) was used following the program and procedures provided by the manufacturer. All primer sets were synthesized commercially by Mission Biotech, Taiwan.

## Supporting Information

Figure S1 **Survival of N3 treated hSCARB2-transgenic mice preinfected with CVA16.** Seven-day old mice preinfected with 1×10^5^ pfu of CVA16 were given 200 µg N3 (•), isotype antibody (♦) and without treatment (▴) at 3 h post infection. Mice were monitored daily and survival rates were recorded. Each group consisted of 5 to 7 mice and the results were representative of 2 independent experiments. The Logrank test was used for statistical analysis.
(TIF)

Figure S2 **N3-mediated neutralization against CVA16 in vitro.** (**A**) CVA16 was pre-incubated (m.o.i. = 10^−3^) with various amounts of N3 for 1 h at 37 ^ο^C before adding them to 3T3-SCARB2 cells. RNA was extracted 2 h after infection, and subjected to RT-PCR to detect the expression of viral genome P1. The amounts of N3 in different lanes were Lane 1∶50 µg, Lane 2∶10 µg, Lane 3∶2 µg, Lane 4∶0.4 µg, Lane 5: infected 3T3-SCARB2 cells without N3, Lane 6: un-infected 3T3-SCARB2 cells as the negative control and Lane 7: CVA16 viral cDNA as the positive control were included. Expression of cytosolic GADPH as the internal control of RT-PCR was detected. (**B)** The bar graph represents the densitometric quantification of the band intensities of viral genome VP1 and GADPH from (**A**) and (**B**), respectively.
(TIF)

Figure S3 **Detection of N3 and mouse anti-CVA16 antisera on different naïve EV71 and CVA16 virions.** The optical density reading of N3 against naïve EV71 genotypes showed high affinity on E59 which was used to raise the antibody. The affinities of N3 on EV71 5746 and CVA16 were reduced. However mouse anti-CVA16 antisera showed similar affinity on detection of these virions. Isotype antibody was also included as background control.
(TIF)

Figure S4 **Protein analysis profiles of N3 and mouse anti-CVA16 on different EV71 genotypes and CVA16.** Purified and concentrated EV71 and CVA16 were separated by SDS-PAGE and transferred to PVDF membrane for Western blotting with N3 (**A**), mouse anti-CVA16 antisera (**B**) or isotype antibody (**C**). (**A**) N3 detected VP1 proteins at approximately 35 kDa from both genotypes of EV71 [17], [37] and the VP0 of CVA16 at approximately 50 kDa [39]. (**B**) anti-CVA16 antisera detected similar capsid protein patterns among both EV71 and CVA16 isolates with highlighted VP3 at approximately 26 kDa. (**C**) Isotype antibody did not detect any protein from both EV71 genotypes and CVA16.
(TIF)
