# Supplementary figures and images for: Protective Efficacy of VP1-Specific Neutralizing Antibody Associated with a Reduction of Viral Load and Pro-Inflammatory Cytokines in Human SCARB2-Transgenic Mice
Source: PLoS One. 2013 Jul 30;8(7):e69858. doi: 10.1371/journal.pone.0069858 (PMC3728341; doi:10.1371/journal.pone.0069858)

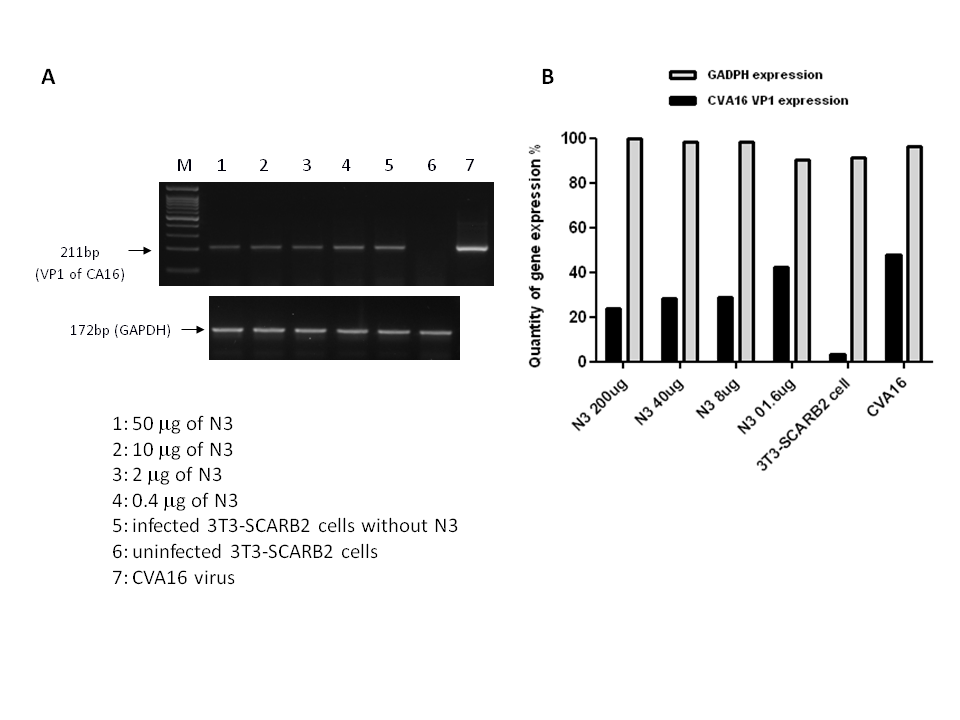

Supplement: Figure S2 — N3-mediated neutralization against CVA16 in vitro. (A) CVA16 was pre-incubated (m.o.i. = 10−3) with various amounts of N3 for 1 h at 37 οC before adding them to 3T3-SCARB2 cells. RNA was extracted 2 h after infection, and subjected to RT-PCR to detect the expression of viral genome P1. The amounts of N3 in different lanes were Lane 1∶50 µg, Lane 2∶10 µg, Lane 3∶2 µg, Lane 4∶0.4 µg, Lane 5: infected 3T3-SCARB2 cells without N3, Lane 6: un-infected 3T3-SCARB2 cells as the negative control and Lane 7: CVA16 viral cDNA as the positive control were included. Expression of cytosolic GADPH as the internal control of RT-PCR was detected. (B) The bar graph represents the densitometric quantification of the band intensities of viral genome VP1 and GADPH from (A) and (B), respectively. (TIF) [file pone.0069858.s002.tif]
